# Supplementary material for: Polarity reversal of stable microtubules during neuronal development
Source: J Cell Sci. 2025 Nov 27;138(22):jcs264152. doi: 10.1242/jcs.264152 (PMC12718633; doi:10.1242/jcs.264152)
Supplement: Supplementary information [file joces-138-264152-s1.pdf]

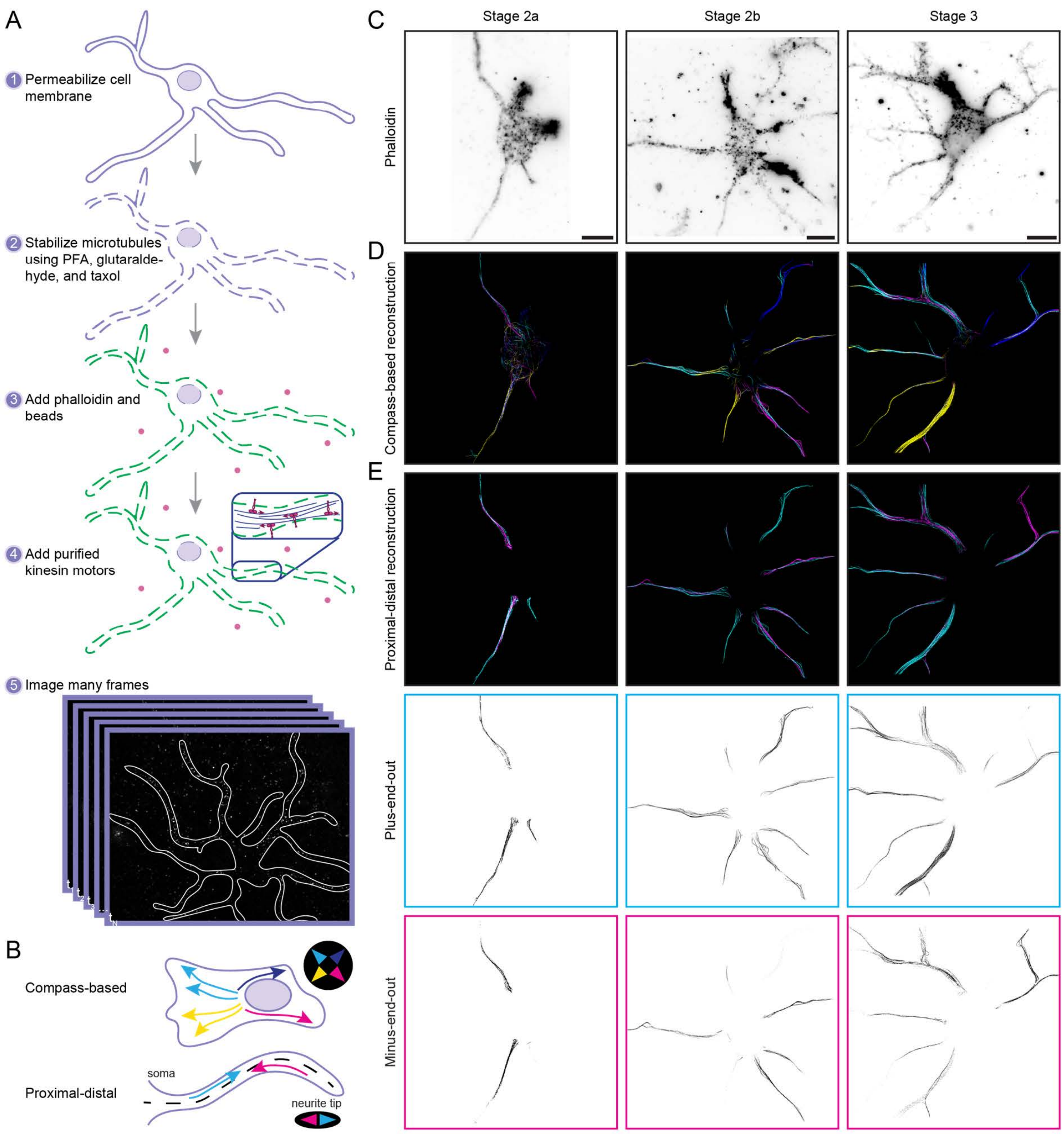

Fig. S1. (A) Schematic showing the motor-PAINT workflow. Neurons are briefly permeabilized (1) and then exposed to low concentrations of PFA and glutaraldehyde for a gentle fixation (2). After washing with a Taxol-containing buffer, neurons are briefly incubated with phalloidin and multicolour beads (3). Subsequently, purified truncated kinesin motors labeled with JF646 are added in an imaging buffer (4), the chamber is sealed, and each cell is imaged for many thousands of frames (5) to allow super-resolutions reconstructions of the microtubules and their orientations. **(B)** Schematic showing the two ways in which tracks of motors can be colour-coded: either by their orientation in the field of view (i.e., towards the top-left, top-right, bottom-left, or bottom-right; compass-based) or by whether they move towards or away from the soma along the neurite (proximal-distal). (C-E) Different views of the same cells shown in Figure 1C. **(C)** Phalloidin stainings of these neurons showing neuron morphology as used to classify cells into stage 2a, 2b or 3. Scale bars 10 µm. **(D)** The total tracks after filtering showing the microtubules in these neurons, but colour-coded based on whether the tracks move towards the top-left, (cyan), top-right (blue), bottom-right (magenta) or bottom-left (yellow). **(E)** The tracks in each of the neurites of these neurons colour-coded based on whether they are moving towards (magenta) or away from (cyan) the soma. Single-channel images are shown below.

Journal of Cell Science • Supplementary information

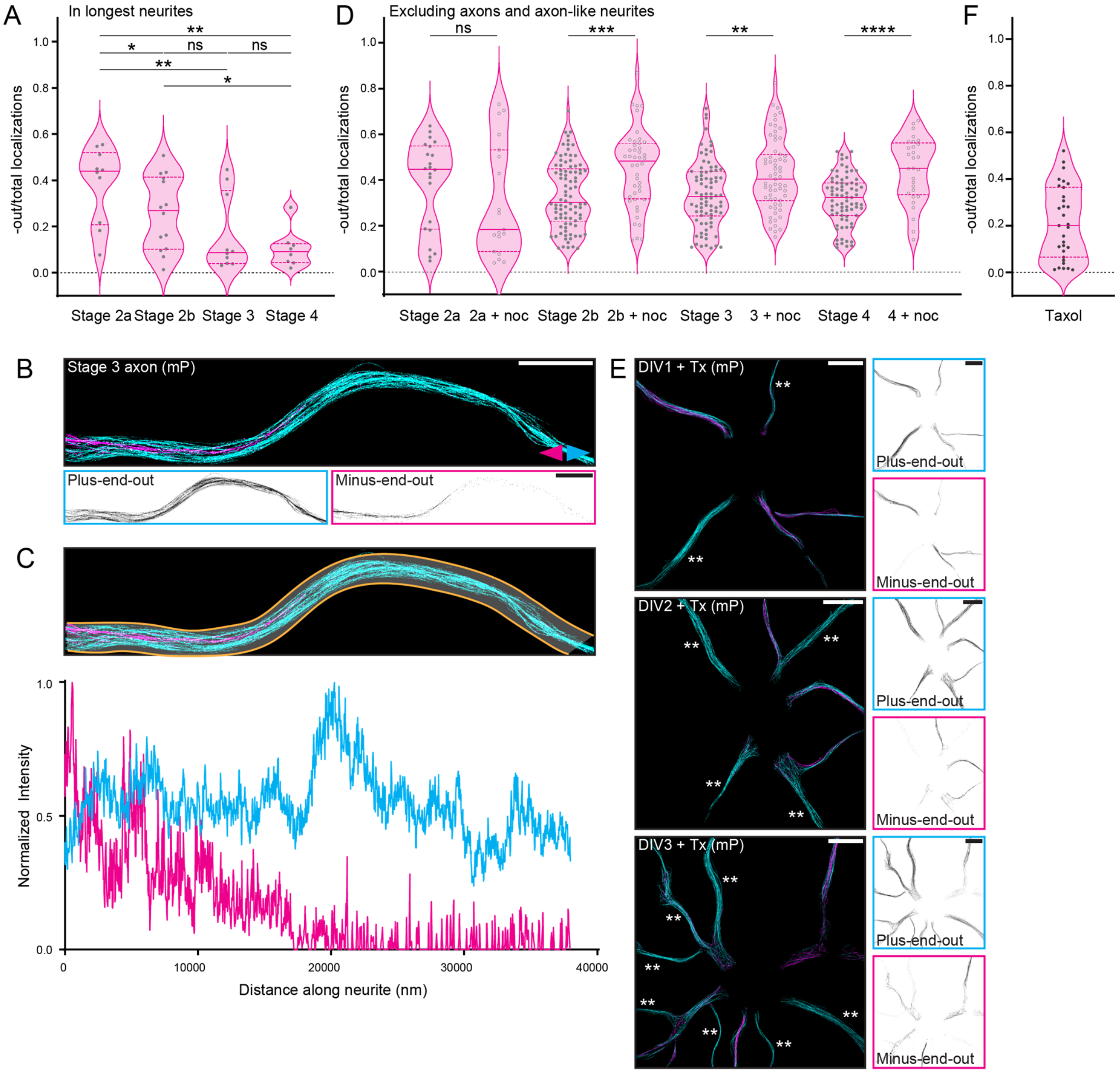

Fig. S2. (A) Summary of the data for cells in which the longest neurite could be clearly identified. Quantification of the fraction of localizations constituting minus-end-out tracks over the total amount of localizations for the longest neurites/axons from neurons in stages 2a, 2b, 3, and 4. Each dot represents one neurite. Medians (0.44, 0.27, 0.09, 0.09) and interquartile ranges ((0.21, 0.52), (0.10, 0.41), (0.04, 0.36), (0.04, 0.13)) are shown. n = 10, 14, 10, 8 neurites from N = 10, 14, 10, 8 cells for stages 2a, 2b, 3, 4. Groups compared by Mann-Whitney test. ns not significant, * $p \leq 0.05$, ** $p \leq 0.01$. (B, C) Some axon-like neurites and axons in stage 2b and stage 3 cells retain a proximal bundle of minus-end-out microtubules. **(B)** A motor-PAINT (mP) reconstruction of an axon/axon-like neurite from a stage 3 cell with microtubules pseudo-coloured based on whether their plus-end is oriented towards (magenta) or away from (cyan) the soma.

Journal of Cell Science • Supplementary information

Single channel images are shown below. Scale bars 2 µm. (C) Intensity profiles of minus-end-out (magenta) and plus-end-out (cyan) microtubules along a line thick enough to enclose the entire neurite (between the yellow lines shown along the neurite). Normalization was done independently for the two orientations using the minimum and maximum values of those data sets. (D) Summary of the data throughout excluding axons and axon-like neurites (defined as a neurite with <10% minus-end-out localizations). Quantification of the fraction of localizations constituting minus-end-out tracks over the total amount of localizations for neurons in stages 2a, 2b, 3, and 4 without and with nocodazole. Each dot represents one neurite. Medians (0.45, 0.18, 0.30, 0.48, 0.33, 0.40, 0.32, 0.45) and interquartile ranges ((0.19, 0.55), (0.09, 0.53), (0.22, 0.45), (0.32, 0.56), (0.24, 0.44), (0.31, 0.51), (0.25,0.39), (0.34, 0.56)) are shown. n = 22, 19, 90, 44, 82, 63, 77, 29 neurites from N = 9, 8, 18, 11, 17, 14, 14, 6 cells for stages 2a without/with, 2b without/with, 3 without/with, and 4 without/with nocodazole. Groups compared by Mann-Whitney test. ns not significant, ** $p \leq 0.01$, *** $p \leq 0.001$, **** $p \leq 0.0001$. **(E)** Representative motor-PAINT (mP) reconstructions of neurons treated with 10 nM Taxol on DIV0 and imaged on DIV1, DIV2, and DIV3 (from top to bottom). The tracks in each of the neurites of these neurons colour-coded based on whether they are moving towards (magenta) or away from (cyan) the soma. ** indicates neurites with (almost) exclusively plus-end-out microtubules. Scale bars 10 µm. **(F)** Quantification of the fraction of localizations constituting minus-end-out tracks over the total amount of localizations for neurons treated with Taxol on DIV1, DIV2, and DIV3. Each dot represents one neurite. Median (0.20) and interquartile range (0.07, 0.36) are shown. n = 31 neurites from N = 2, 2, 2 cells for DIV1, DIV2, DIV3.

Journal of Cell Science • Supplementary information

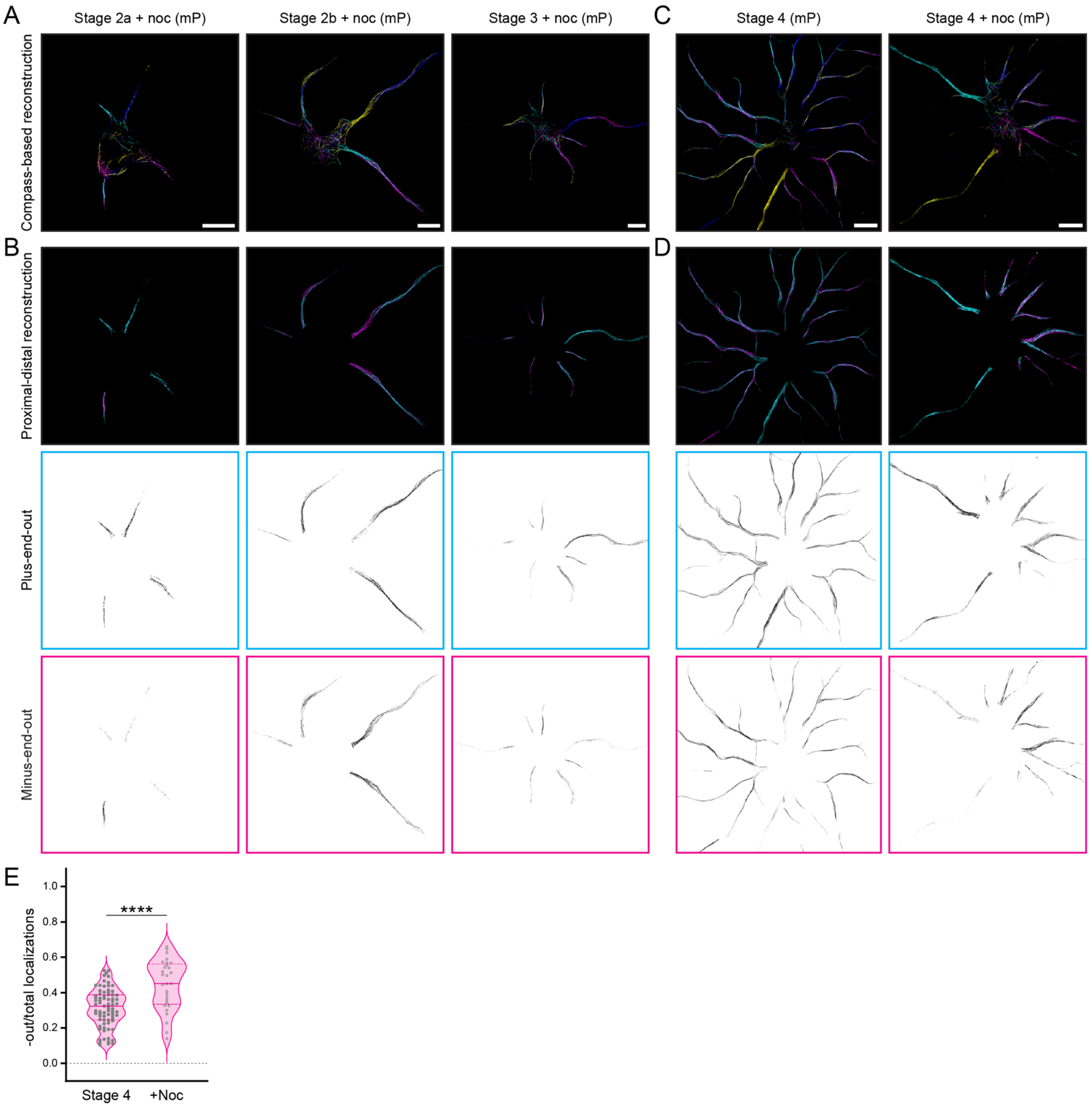

Fig. S3. Extra views of neurons showing the orientation of microtubules remaining after nocodazole treatment. **(A)** The total tracks after filtering showing the microtubules in the neurons shown in Figures 3B, D, and F imaged by motor-PAINT (mP), but colour-coded based on whether the tracks move towards the top-left, (cyan), top-right (blue), bottom-right (magenta) or bottom-left (yellow). Scale bars 10 µm. **(B)** The tracks in each of the neurites of these neurons colour-coded based on whether they are moving towards (magenta) or away from (cyan) the soma. Single-channel images are shown below. **(C)** The total tracks after filtering showing the microtubules in a stage 4 neuron without (left) and with (right) nocodazole imaged by motor-PAINT (mP), but colour-coded

based on whether the tracks move towards the top-left, (cyan), top-right (blue), bottom-right (magenta) or bottom-left (yellow). Scale bars 10 µm. **(D)** The tracks in each of the neurites of these neurons colour-coded based on whether they are moving towards (magenta) or away from (cyan) the soma. Single-channel images are shown below. **(E)** Quantification of the fraction of localizations constituting minus-end-out tracks over the total amount of localizations for stage 4 neurons without and with (+Noc) a nocodazole treatment. Each dot represents one neurite. Medians (0.28, 0.45) and interquartile ranges ((0.13, 0.37), (0.31, 0.55)) are shown. n = 94, 32 neurites from N = 14, 6 cells for control and nocodazole-treated cells. Groups compared by Mann-Whitney test. **** $p \leq 0.0001$.

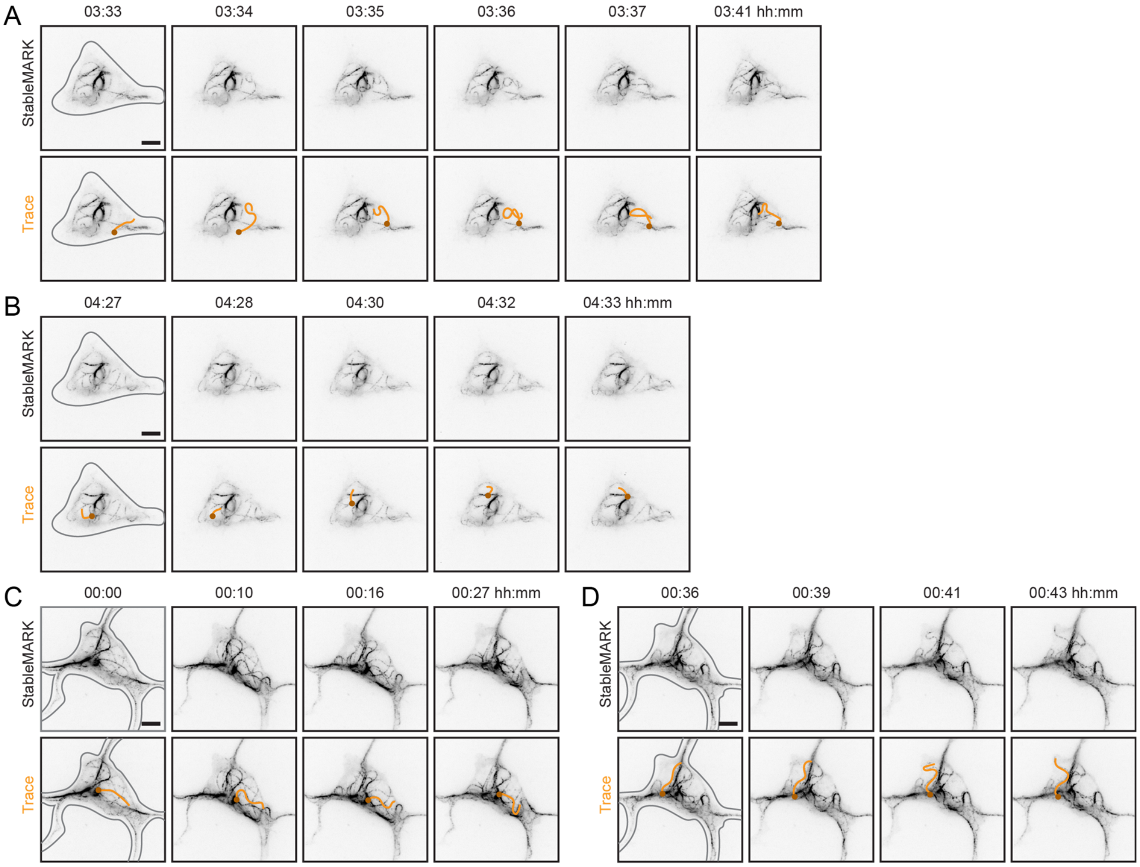

Fig. S4. Live cell examples showing the highly dynamic stable microtubules in stage1/stage 2a (**A**, **B**) and stage 2b (**C**, **D**) neurons. Neurons were electroporated with StableMARK. Example microtubules are traced in orange in each of the bottom rows, with the same end marked by a small circle in each frame to indicate orientation. First frames also have the cell outline in grey. Times shown above each frame are in hours:minutes. Scale bars 5 µm. A and B correspond to Movie 6. C and D correspond to Movie 7.

Journal of Cell Science • Supplementary information

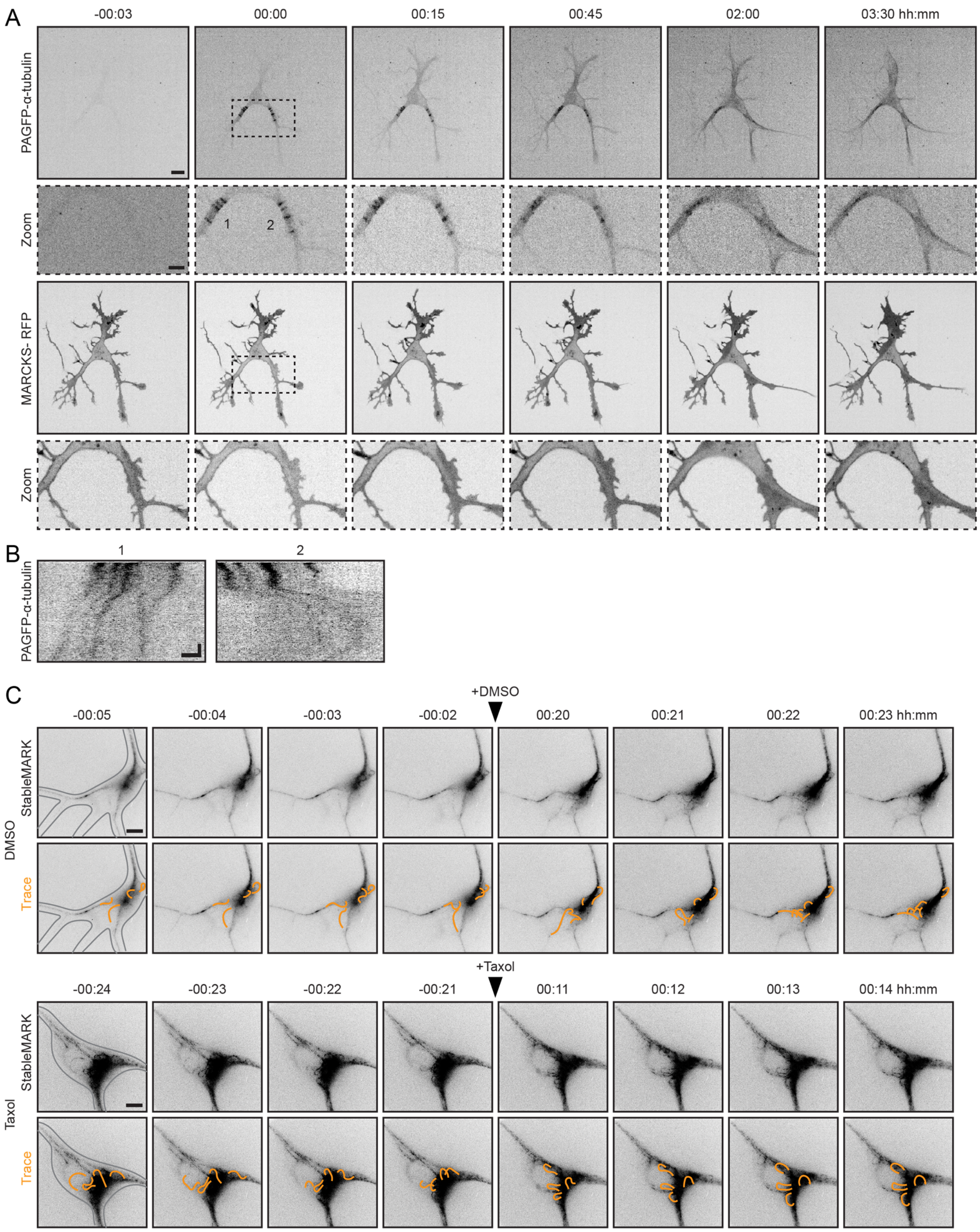

Fig. S5. (A) Live cell data confirming the long lifetime of stable microtubules using photoactivatable GFP (PAGFP)-α-tubulin and MARCKS-RFP (to show the cell membrane). Scale bars 10 μm whole cell, 5 μm zooms. **(B)** Kymographs corresponding to the neurites labelled as 1 and 2 in (A) shown proximal to distal (left-to-right). The photoconverted regions can be followed for many hours suggesting this tubulin remains in the same microtubules. Scale bars 2 μm (horizontal) and 30 min (vertical). **(C)** Live-cell data showing the change in motility of stable microtubules following the addition of DMSO (top) or Taxol (bottom). Example microtubules are traced in orange in each of the bottom rows. First frames also have the cell outline in grey. Times shown above each frame are in hours:minutes. Scale bars 5 μm.

Journal of Cell Science • Supplementary information

Table S1. Antibody sources, dilutions and applications

**Antibody**	**Company**	**Catalog No.**	**Dilution**	**Use**
Rat anti-tyrosinated tubulin	Abcam	ab6160	1:500<br>1:250	Confocal<br>U-ExM
Rabbit anti-acetylated tubulin	Cell Signaling Technology	5335	1:500<br>1:250	Confocal<br>U-ExM
Alexa Fluor 647 Goat anti-rat	Life Technologies	A-21247	1:500	Confocal
Alexa Fluor 488 Goat anti-rabbit	Thermo Fisher Scientific	A-11008	1:500<br>1:500	Confocal<br>U-ExM
Alexa Fluor 594 Goat anti-rat	Thermo Fisher Scientific	A-11007	1:500	U-ExM

Journal of Cell Science • Supplementary information

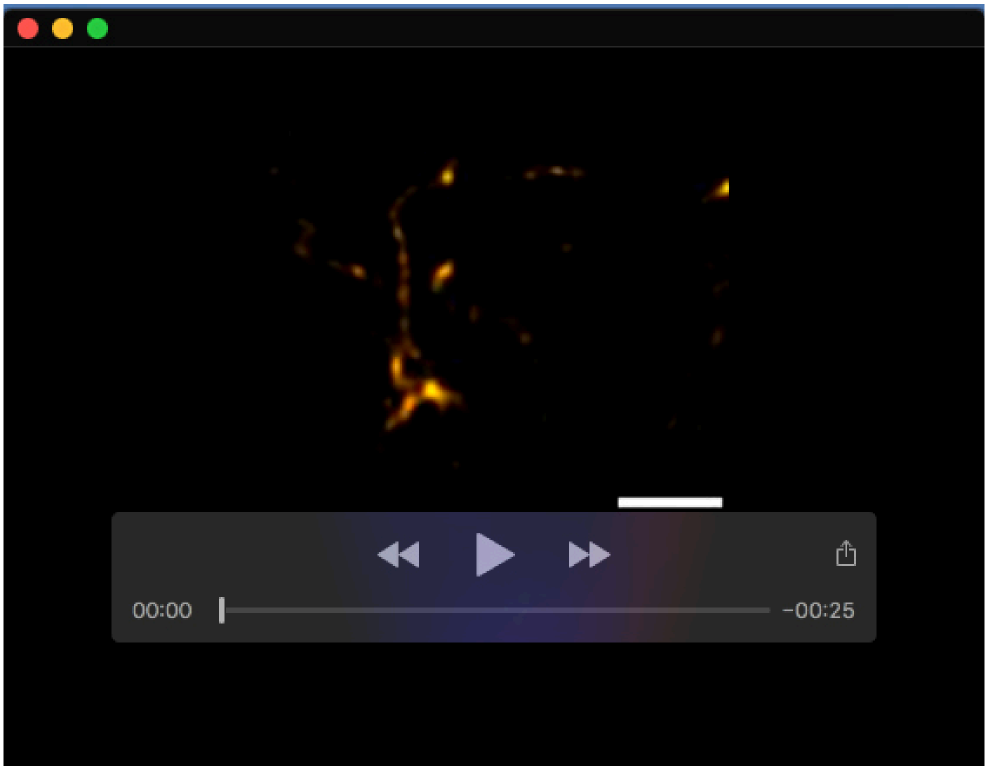

Movie 1. An example of the acetylated microtubules around the centrioles of a stage 1 neuron showing. Movie played at 7 fps. Scale bar 500 nm (corrected for expansion factor).

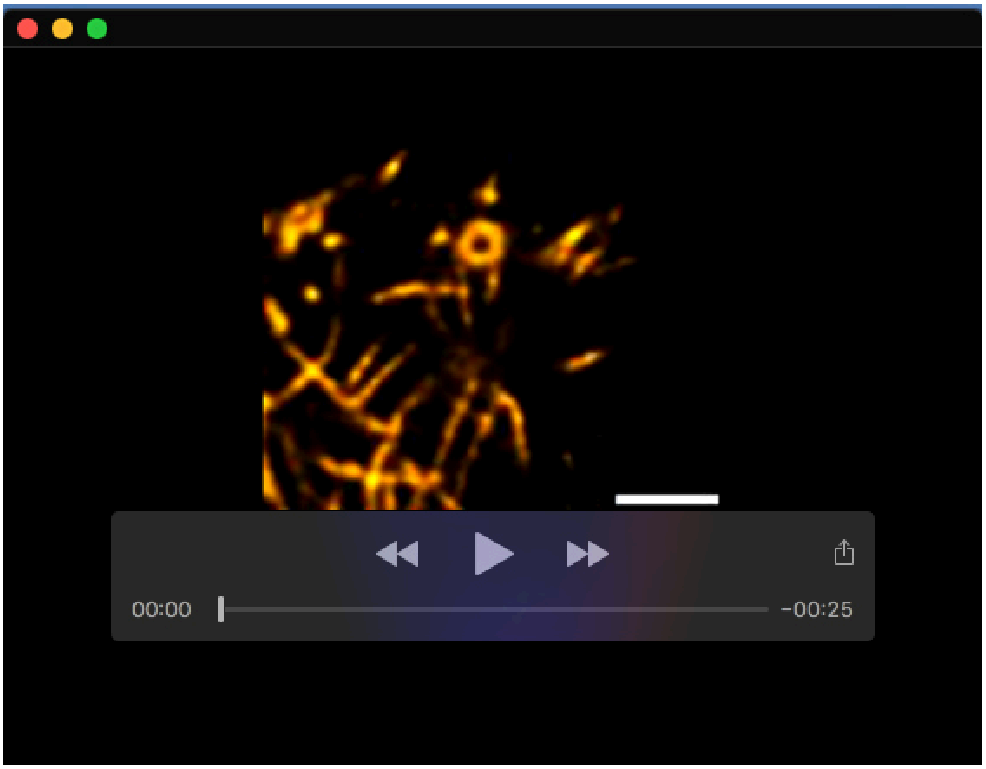

Movie 2. An example of the acetylated microtubules around the centrioles of a stage 2a neuron showing. Movie played at 7 fps. Scale bar 500 nm (corrected for expansion factor).

Journal of Cell Science • Supplementary information

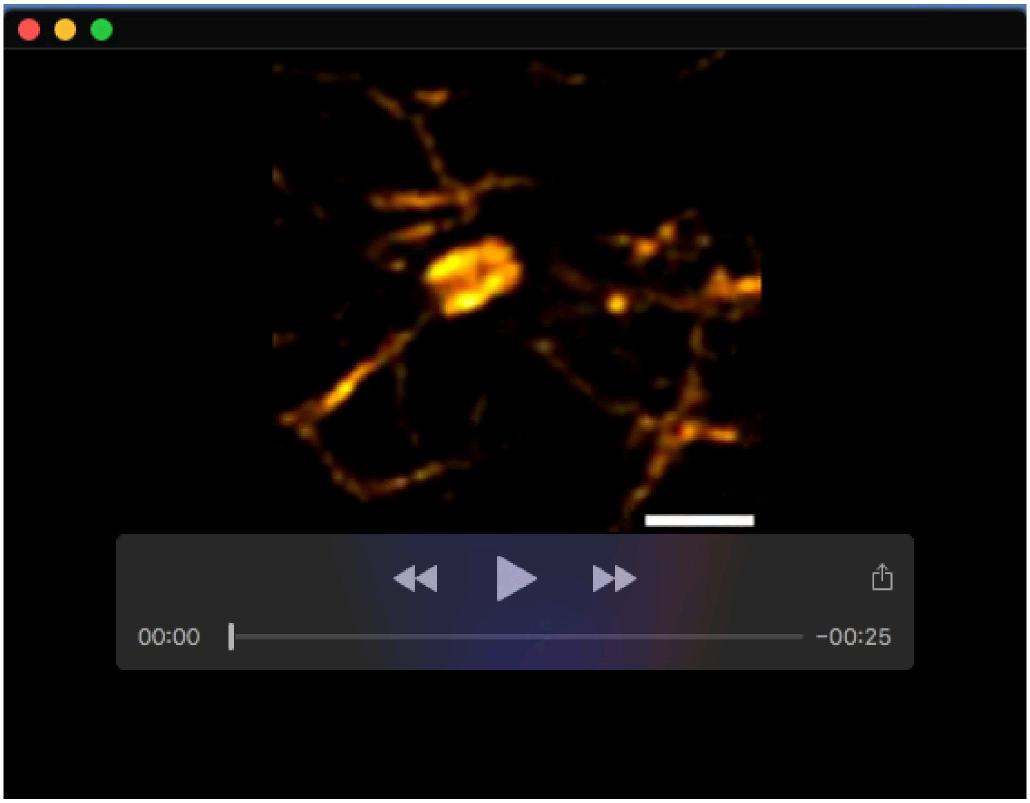

Movie 3. An example of the acetylated microtubules around the centrioles of a stage 2b neuron showing. Movie played at 7 fps. Scale bar 500 nm (corrected for expansion factor).

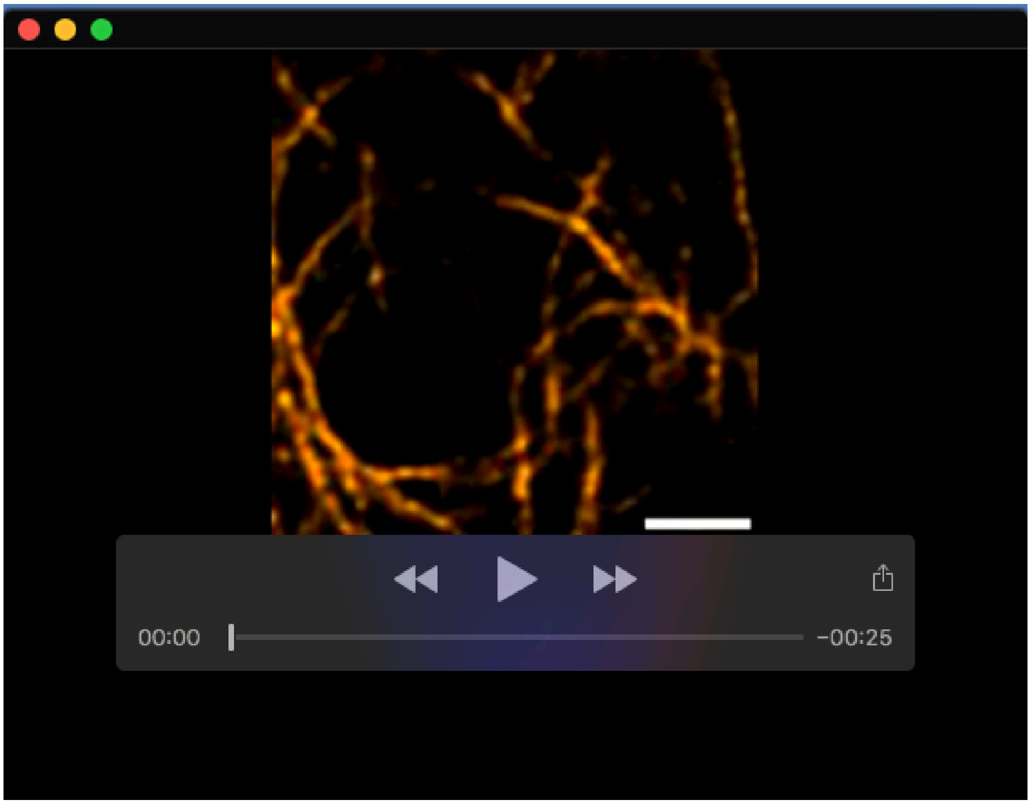

Movie 4. An example of the acetylated microtubules around the centrioles of a stage 3 neuron showing. Movie played at 7 fps. Scale bar 500 nm (corrected for expansion factor).

Journal of Cell Science • Supplementary information

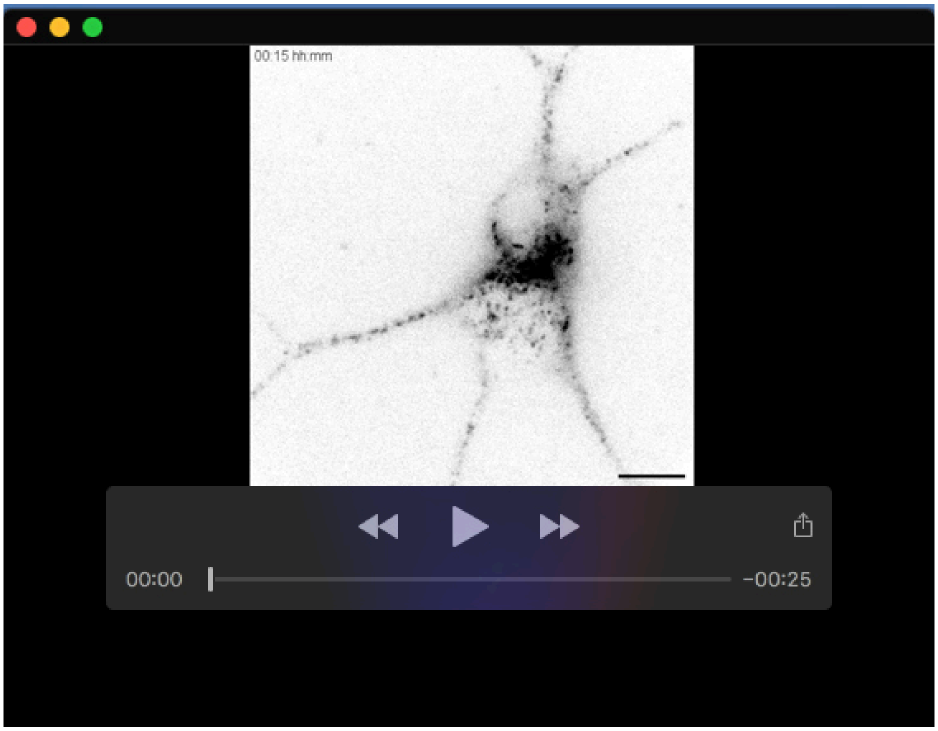

Movie 5. An example of a stage 2b neuron expressing StableMARK showing the retrograde flow of stable microtubules in the neurites. Timestamps shown are in hours:minutes. Movie played at 10 fps. Scale bar 5 µm.

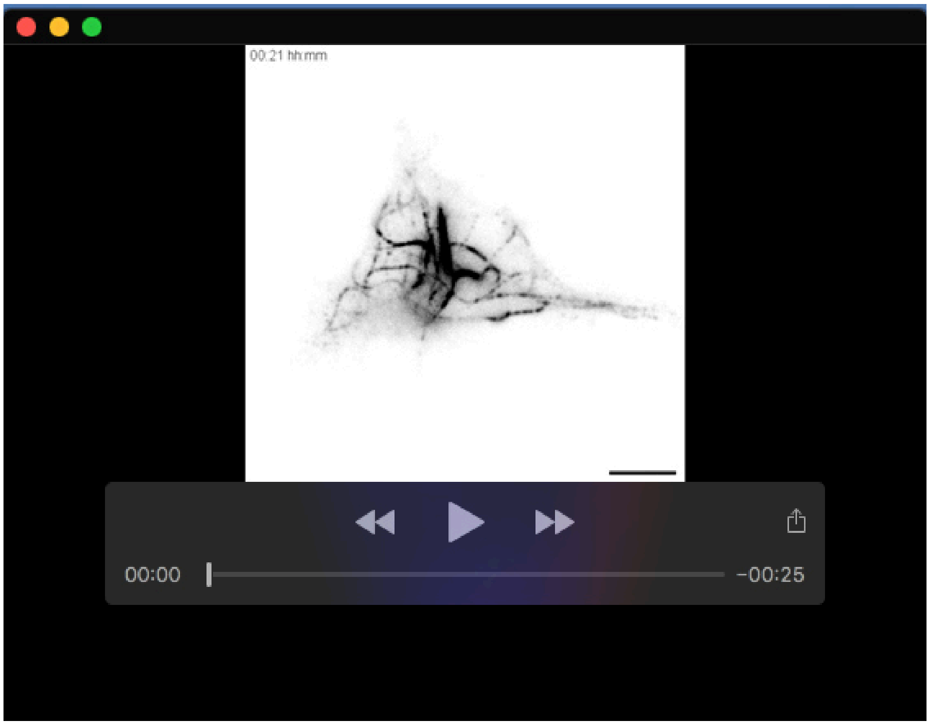

Movie 6. An example of a stage 2a neuron expressing StableMARK showing the very motile and sliding stable microtubules in this stage. Timestamps shown are in hours:minutes. Movie played at 10 fps. Scale bar 5 µm.

Journal of Cell Science • Supplementary information

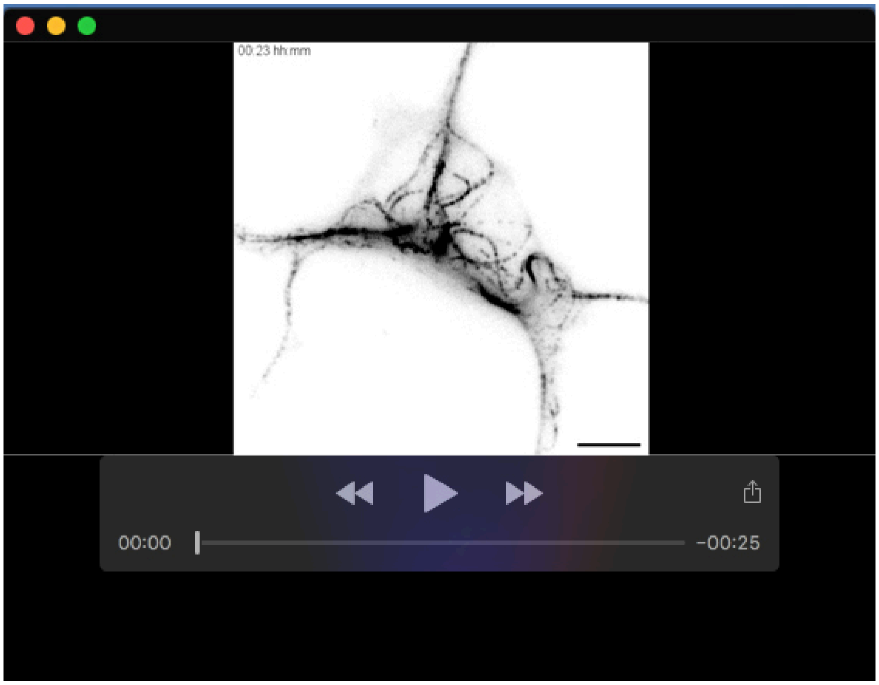

Movie 7. An example of a stage 2b neuron expressing StableMARK showing the very motile and sliding stable microtubules in this stage, as well as the clear link of a bundle of stable microtubules to a focal point (potentially the centrosome). Also shows curling back of stable microtubules in neurites to reverse orientation. Timestamps shown are in hours:minutes. Movie played at 10 fps. Scale bar 5 µm.

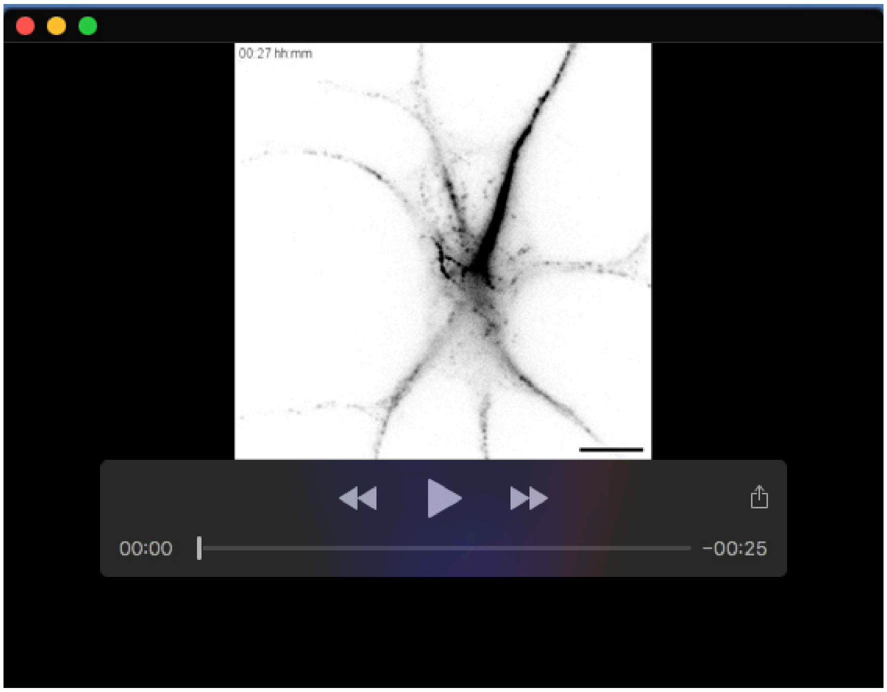

Movie 8. An example of a stage 2b neuron expressing StableMARK showing the very stable link of a bundle of stable microtubules to the centrosome. Timestamps shown are in hours:minutes. Movie played at 10 fps. Scale bar 5 µm.

Journal of Cell Science • Supplementary information

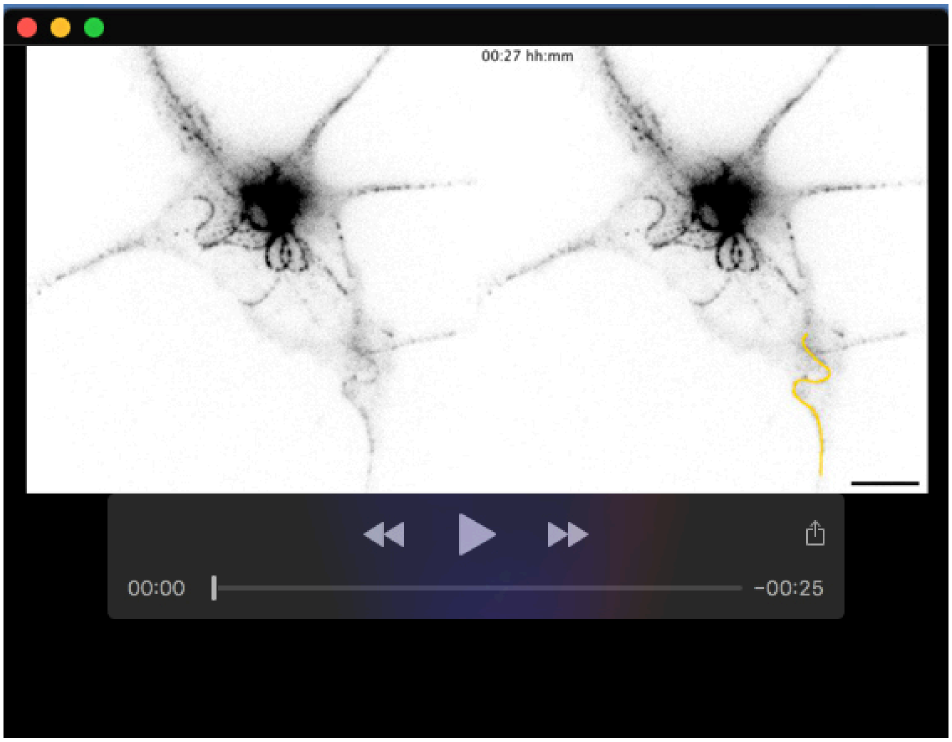

Movie 9. An example of a stage 2b neuron expressing StableMARK showing two means by which stable microtubules can reverse their orientation: sliding from one neurite into another (also traced in orange) and reversing within the same neurite (also traced in blue). Timestamps shown are in hours:minutes. Movie played at 10 fps. Scale bar 5 µm.

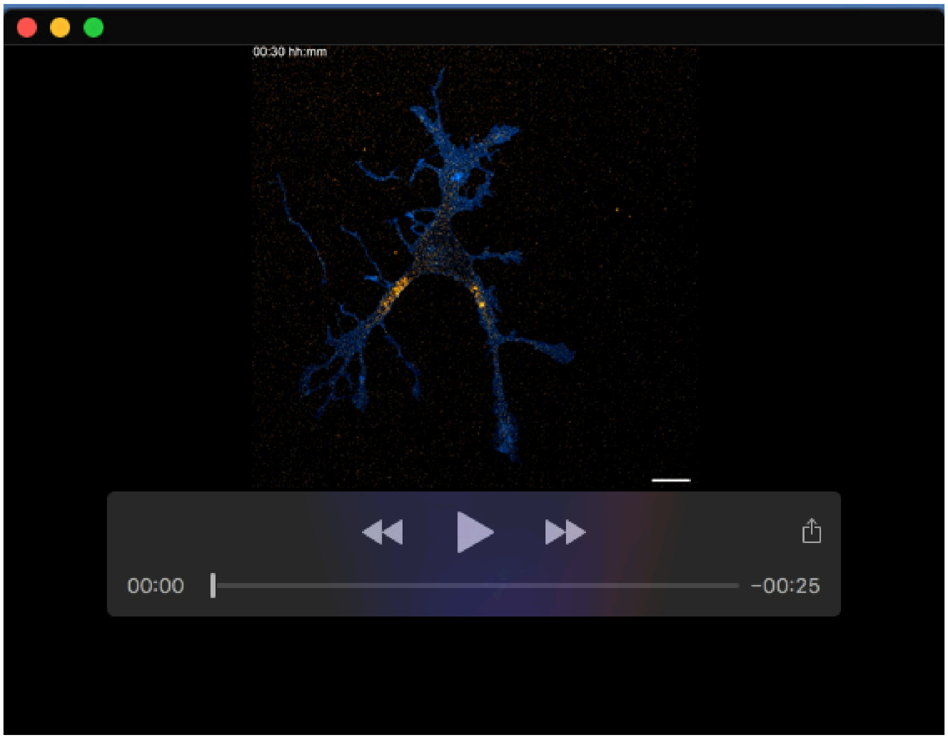

Movie 10. An example of a stage 2b neuron expressing photoactivatable GFP-α-tubulin (orange) and MARCKS-RFP (blue). Note the persistence of the GFP signal over time because the microtubules with the photoactivated GFP-α-tubulin persist even as they slide around. Timestamps shown are in hours:minutes. Movie played at 7 fps. Scale bar 10 µm.

Journal of Cell Science • Supplementary information

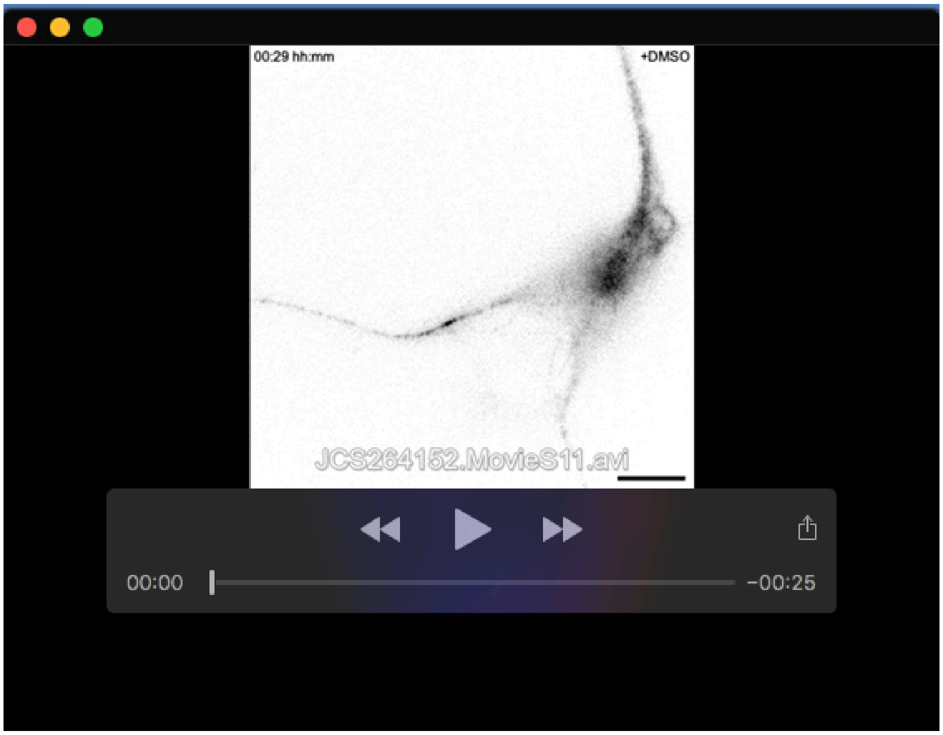

Movie 11. An example of a stage 2b neuron expressing StableMARK to which DMSO is added after 30 minutes, showing no change in microtubule motility. Timestamps shown are in hours:minutes. Movie played at 10 fps. Scale bar 5 µm.

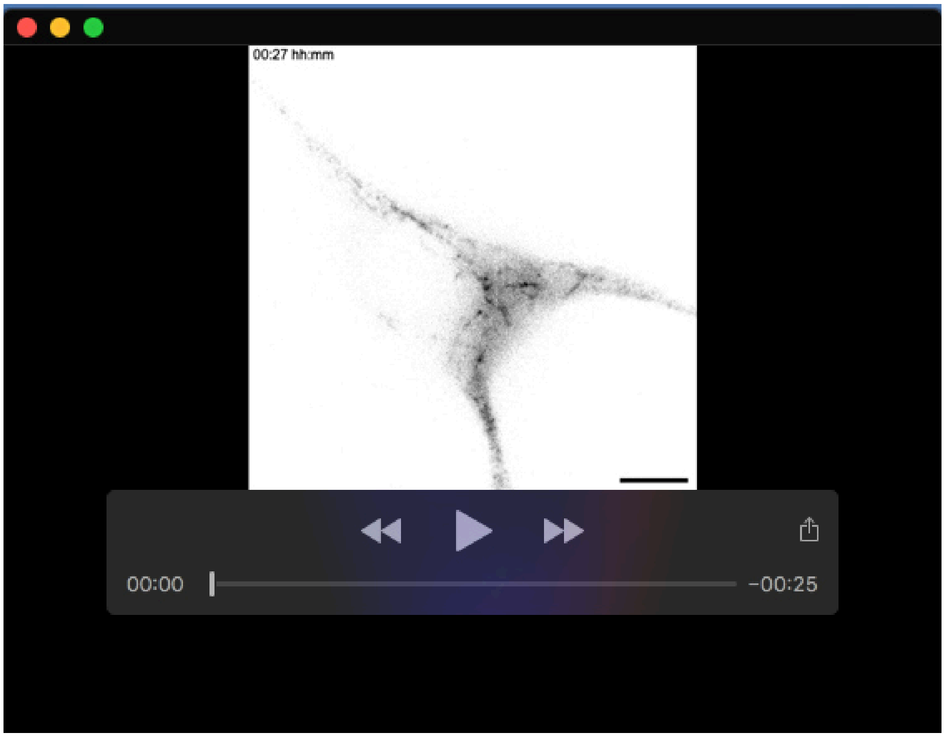

Movie 12. An example of a stage 2b neuron expressing StableMARK to which Taxol is added after 30 minutes showing a reduction in microtubule motility. Timestamps shown are in hours:minutes. Movie played at 10 fps. Scale bar 5 µm.

Journal of Cell Science • Supplementary information
